# The Multiple Hit Hypothesis for Gulf War Illness: Self-Reported Chemical/Biological Weapons Exposure and Mild Traumatic Brain Injury

**DOI:** 10.3390/brainsci8110198

**Published:** 2018-11-13

**Authors:** Patricia Janulewicz, Maxine Krengel, Emily Quinn, Timothy Heeren, Rosemary Toomey, Ronald Killiany, Clara Zundel, Joy Ajama, James O’Callaghan, Lea Steele, Nancy Klimas, Kimberly Sullivan

**Affiliations:** 1 Department of Environmental Health, Boston University School of Public Health, Boston, MA 02118, USA; ajamajoy@bu.edu (J.A.); tty@bu.edu (K.S.); 2 Research Service, VA Boston Healthcare System, Boston, MA 02130, USA; mhk@bu.edu; 3 Department of Neurology, Boston University School of Medicine, Boston, MA 02118, USA; 4 Biostatistics and Epidemiology Data Analytics Center, Boston University School of Public Health, Boston, MA 02118, USA; eq@bu.edu; 5 Department of Biostatistics, Boston University School of Public Health, Boston, MA 02118, USA; tch@bu.edu; 6 Department of Psychological and Brain Sciences, Boston University, Boston, MA 02215, USA; toomey@bu.edu; 7 Department of Anatomy and Neurobiology, Boston University School of Medicine, Boston, MA 02118, USA; killiany@bu.edu; 8 Department of Behavioral Neuroscience, Boston University School of Medicine, Boston, MA 02118, USA; cgzundel@bu.edu; 9 Centers for Disease Control and Prevention-NIOSH, Morgantown, WV 26505, USA; jdo5@cdc.gov; 10 Department of Psychiatry and Behavioral Sciences, Division of Neuropsychiatry, Baylor College of Medicine, Houston, TX 77030, USA; Lea.Steele@bcm.edu; 11 Department of Medicine, Miami VA Healthcare System, Miami, FL 33125, USA; nklimas@nova.edu; 12 Department of Clinical Immunology, Nova Southeastern University, Miami, FL 33314, USA

**Keywords:** Gulf War Illness, Gulf War, mild traumatic brain injury, chemical weapons, neurotoxicant exposures

## Abstract

The Gulf War Illness Consortium (GWIC) was designed to identify objective biomarkers of Gulf War Illness (GWI) in 1991 Gulf War veterans. The symptoms of GWI include fatigue, pain, cognitive problems, gastrointestinal, respiratory, and skin problems. Neurotoxicant exposures during deployment, such as pesticides, sarin, and pyridostigmine bromide pills have been identified as contributors to GWI. We have also found an association between mild traumatic brain injury (mTBI) and increased rates of GWI. However, the combined impact of these physical and chemical exposures has not yet been explored in GWI. The objective of this study was to examine both self-reported mTBI and exposure to chemical/biological weapons (CBW) as a multiple or two hit model for increased risk of GWI and other chronic health conditions. The study population included 125 Gulf War (GW) veterans from the Boston GWIC. Exposure to CBW was reported in 47.2% of the study population, and 35.2% reported sustaining a mTBI during the war. Results confirmed that those with both exposures (mTBI and CBW) had higher rates of comorbid chronic health conditions while rates of GWI were equivalent for mTBI and CBW or mTBI alone. The timing of exposure to mTBI was found to be strikingly different between those with GWI and those without it. Correspondingly, 42.3% of GWI cases reported experiencing a mTBI during military service while none of the controls did (*p* = 0.0002). Rates of mTBI before and after the war did not differ between the cases and controls. In addition, 54% of cases compared to 14.3% of controls (*p* = <0.001) reported being exposed to CBW during military service. The current study examined the relation of the separate and combined effects of exposure to mTBI and CBW in 1991 GW veterans. The findings from this study suggest that both exposure to mTBI and CBW are associated with the development of GWI and multiple chronic health conditions and that combined exposure appears to lead to higher risk of chronic health effects.

## 1. Introduction

Exposure to chemical mixtures has long been reported as a potential cause for Gulf War (GW) veterans’ chronic health symptoms [1,2,3,4,5]. It has been found in numerous studies that GW veterans with exposure to neurotoxicants, including pesticides, pyridostigmine bromide pills, and chemical/biological weapons (CBW), have higher rates of chronic symptoms than those without such exposures [6]. In fact, a recent study found a strong inverse correlation with veterans reporting total number of chemical alarm sounds heard during the war and total cortical gray matter brain volumes [7]. Additionally, GW veterans with an increased reporting of CBW exposures have more frequently met criteria for the current GW Illness case definitions [8], including chronic multi-symptom illness (CMI) [9] and Kansas GWI criteria [10], than those without such exposures [11,12,13]. The symptoms that have most often been found to correlate with CBW exposures have been fatigue, muscle aches, irritability, cognitive impairment, and a depressed and anxious mood. These symptoms notably engulf multiple organ systems that are reflective of central nervous system (CNS) impairment [2,6,14,15].

Mild traumatic brain injury (mTBI) has been reported as another less intuitive potential factor in the development of chronic health symptoms experienced by GW veterans that can also affect CNS functioning [16,17]. Correspondingly, GW veterans with any history of mTBI in the large, longitudinally followed Ft. Devens cohort (FDC) have been found to have significantly higher rates of GWI (Kansas and CMI criteria) than those without mTBI [16,17]. In addition, the total number of health symptoms reported by the Ft. Devens cohort was positively correlated with total number of mTBIs. These symptoms included CNS and non-CNS body systems. Correspondingly, FDC studies also have shown differences in veterans with mTBI versus no mTBI in reported health symptoms across multiple functional domains, including cardiac, dermatological, gastrointestinal, musculoskeletal, neurological, neuropsychological, and pulmonary [17]. In another smaller cohort, Chao et al. [18] reported that a history of mTBI was not associated with higher rates of GWI when Kansas criteria or severe CMI criteria were used, but rather only when mild or moderate CMI criteria were used. This study also found that the highest rates of mTBI were reported before the war and after the war, with the lowest rates occurring during the war. However, neither the FDC nor the Chao study assessed the combined impact of mTBI and CBW exposures during the war on the development of chronic GWI and other medical outcomes. The results of these two sets of studies raise important questions about the impact of timing of the mTBI on chronic health symptoms and the development of chronic GWI symptoms.

Given that both mTBI and CBW have the capacity to influence CNS function, it is possible that those who have experienced both “exposures” may have higher rates of chronic symptoms than those with only one or neither as part of a “two hit” or “multiple hit” scenario. What has been lacking in the literature on GWI is the relative impact of these multiple exposures (e.g., TBI and CBW) on health symptoms and the documentation as to whether or not there is an interaction of the two in terms of chronicity of health outcomes. Therefore, the purpose of this study was to document a “multiple hit hypothesis” in GWI. Multiple hit hypotheses investigate the effects of multiple risk factors or insults, additive or interactive, on an outcome. Numerous studies have utilized the multiple hit hypothesis to investigate nervous system insults on various outcome measures [19,20,21,22,23]. The current study compared multiple GW exposures (mTBI and CBW) in a more representative sample of GW veterans than those mentioned above from different parts of the country, representing all branches of the military and with varying occupational specialties during the war.

It was hypothesized that GW veterans who had both a history of mTBI and CBW exposures would have increased rates of GWI and other chronic health outcomes compared to GW veterans who had only a history of mTBI or only CBW toxicant exposure. GW veterans with multiple “hits” during the war would be at significantly greater risk for illness than those with no “hits” during the war. It was also hypothesized that GW veterans who had both a history of mTBI and CBW exposures (“two hits”) would have increased rates of health outcomes compared to GW veterans who had only a history of mTBI or only CBW exposure. To our knowledge, this is the first paper to assess the impact of CBW exposure and mTBI during the war on GWI and other chronic health outcomes in GW veterans in a new more representative sample of GW veterans recruited as part of the Boston multi-site Gulf War Illness Consortium (GWIC).

## 2. Materials and Methods

### 2.1. Participants

The study population included 125 GW veterans from the Boston University Gulf War Illness Consortium (GWIC). The GWIC is a multi-site consortium with three clinical sites around the country (Boston, Miami, and Houston). The inclusion criteria required deployment to the Gulf War between August 1990 and July 1991. The exclusion criteria for the study included diagnoses that could otherwise account for the symptoms reported by the veterans. This included autoimmune, central nervous system, or major psychiatric disorders that may affect brain and immune functions (e.g., epilepsy, stroke, severe head injury, brain tumor, multiple sclerosis, Parkinson’s disease, Alzheimer’s disease, schizophrenia, bipolar disorder, and autoimmune disorders). The Kansas GWI case criteria required endorsement of at least 3 out of 6 symptom domains (fatigue, pain, neurological, skin, gastrointestinal, and respiratory) of at least moderate severity. GW veterans not meeting Kansas or exclusionary criteria were considered controls. Once enrolled, subject data were obtained between 2013 and 2018.

### 2.2. Self-Reported Mild Traumatic Brain Injury (mTBI)

To assess the occurrence of mTBI, participants were provided with a definition of concussion consistent with current guidelines from the American Academy of Neurology, which has been used in several recent publications [24,25,26]. This definition clarifies what constitutes a mTBI and provides examples of common symptoms [24]. The following description was provided to the participants;

“Some people have the misconception that mild traumatic brain injury (also known as ‘concussion’) only happens when you lose consciousness after being hit on the head or when the symptoms last for a long time. However, a mild traumatic brain injury occurs anytime you have an impact to the head that causes symptoms for any amount of time (e.g., seconds or longer). These symptoms include: sensitivity to light or noise, headache, dizziness, balance problems, nausea, vomiting, trouble sleeping, fatigue, confusion, difficulty remembering, difficulty concentrating, or loss of consciousness.”

Participants were asked to report whether they had experienced any mTBIs during three time-periods: before the war, during the war, and after the war. In addition, they were asked to self-report how many mTBIs they sustained during each of those periods given this definition and to be sure to include those from participation in sports.

### 2.3. Demographics, Chemical/Biological Weapon (CBW) Exposure, and Health Outcome Surveys

GWIC subjects were administered a general demographic information and medical conditions questionnaire, the Kansas Gulf War and Health Questionnaire and Kansas Gulf War Experiences and Exposure Questionnaire, and the Structured Neurotoxicant Assessment Checklist (SNAC) [6,10,27]. Self-reported exposures were obtained from the SNAC [6]. For the purpose of this study, we analyzed the SNAC self-reported exposure to CBWs by asking whether or not they were exposed during military service. Health outcomes were measured by a medical conditions questionnaire in which the participant endorsed whether or not they had a confirmed diagnosis of any of the 20 queried health outcomes, including asthma, diabetes, respiratory problems, hypertension, memory loss, chronic fatigue syndrome, and irritable bowel syndrome [6].

### 2.4. Clinical Interview

The Clinician Administered Posttraumatic Stress Disorder Scale (CAPS) [28] and Structured Clinical Interview for Diagnostic and Statistical Manual 4 Diagnosis (SCID) [29] were administered to each participant by a trained examiner. The CAPS for Diagnostic and Statistical Manual 4th edition (DSM-IV) asked the participant to describe any traumatic event and to answer direct questions about symptoms relating to intrusions, avoidance, and cognitive and mood alterations secondary to the event. The SCID was also an interview-based survey enquiring about specific symptoms relating to psychiatric diagnoses. For the purpose of this study, the utilized modules included those for major depression, bipolar disorder, and anxiety disorders other than post-traumatic stress disorder (PTSD).

### 2.5. Data Analysis

We evaluated the association between mTBI and CBW exposure and demographic and health outcomes in 125 GWIC study participants. Chi-square and one-way ANOVA tests were used, as appropriate, to compare exposure groups for descriptive purposes. Separate, unadjusted logistic regressions were performed to determine the effect of mTBI, CBW exposure, and simultaneous mTBI and CBW exposure during military service on health outcomes. Unadjusted Odds Ratios (OR) and 95% confidence intervals (CI) from regression models are reported. Analyses were conducted using 2-sided tests and an alpha level of 0.05. All analyses were conducted using SAS 9.4 (Statistical Analysis Systems, Cary, NC, USA).

## 3. Results

### 3.1. Participant Characteristics

The first 125 GWIC participants from three parts of the country made up the study sample. The study sample was 84.8% male with a mean age of 51.5 years. The majority of participants were white (74.4%) and highly educated, with 82.4% of the population having at least a two-year college degree (Table 1). The study sample was representative of all branches of the military. Army was the most common branch of service (67.2%), followed by Navy (16.0%), Marines (10.4%), and then Air Force (6.4%). In this sample of GW veterans, 34 had a current diagnosis of PTSD as determined by the CAPS criteria while 83.2% of the cohort met the Kansas GWI case criteria.

### 3.2. CBW and mTBI Self-Reported Exposures in GWIC Cohort

CBW exposure during military service was reported in 47.2% of the GWIC study population and 35.2% reported sustaining a mTBI during the war. Subjects were also asked to report mTBI during their lifetime including before, during, and after the war. In the overall study population, 36.8% of veterans reported having a mTBI before the war, 35.2% reported experiencing a mTBI during the war, and 26.4% reported having a mTBI after the war (Table 1).

### 3.3. Results of mTBI and CBW Self-Reported Exposure by GWI Case Status

**Figure 1:**
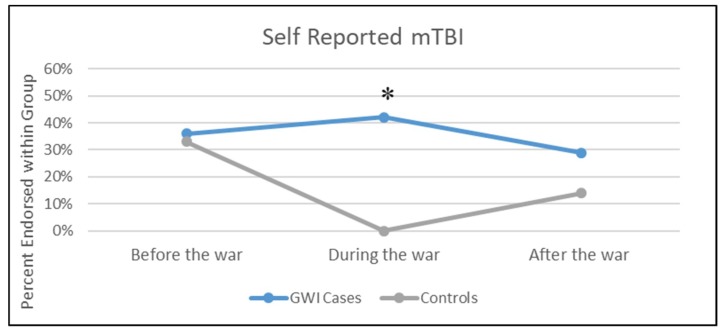
Percent of veterans with self-reported mTBI before, during, and after the war. * *p* < 0.005.

Statistically significant differences were found between the veterans who self-reported mTBI and CBW exposure during military service by GWI case status. GW veterans with GWI were much more likely to have reported experiencing CBW exposure during military service. Fifty-four percent of cases compared to 14.3% of controls (*p* = <0.001) reported being exposed to CBW. In addition, the time-period of exposure was found to be of great importance when examining whether the veterans with GWI experienced any mTBIs. Correspondingly, 42.3% of GWI cases reported experiencing a mTBI during the war while none of the controls reported experiencing a mTBI during the war (*p* = 0.0002) (Figure 1). Thirty-eight percent of GWI cases reported experiencing a mTBI before the war compared to 33.3% of controls (*p* = 0.72). Self-reported mTBI after the war was not found to be statistically different between the two groups (28.9% of cases vs. 14.3% of controls, *p* = 0.17).

### 3.4. Participant Characteristics by Exposure Categories

The study population was classified into four exposure groups based on the following definitions: mTBI and CBW exposure (yes/yes), mTBI exposure and no CBW exposure (yes/no), no mTBI exposure and CBW exposure (no/yes), and lastly no mTBI and no CBW exposure (no/no). Examining the groups in this way allowed us to examine the effect of individual exposure (CBW only and mTBI only) and the multiple exposure (CBW and mTBI) categories. As a result of small numbers, the mTBI variable used in the analysis for this paper was limited to self-reported mTBI during the war (yes/no) and the number of mTBIs was not examined. The number of subjects with GWI significantly differed by mTBI and CBW groups but was not higher for the combined groups compared with mTBI only (Table 2). Specifically, 100% of the mTBI/CBW and mTBI-only groups, 90.9% of the CBW-only group, and 62.5% of the no mTBI/CBW group met the criteria for GWI (*p* < 0.001). In terms of demographic comparisons of the four groups, there was a statistically significant difference between the groups in the number of subjects of Hispanic or Latino descent but no other demographic outcomes differed.

### 3.5. Kansas Criteria by Exposure Group

**Figure 2:**
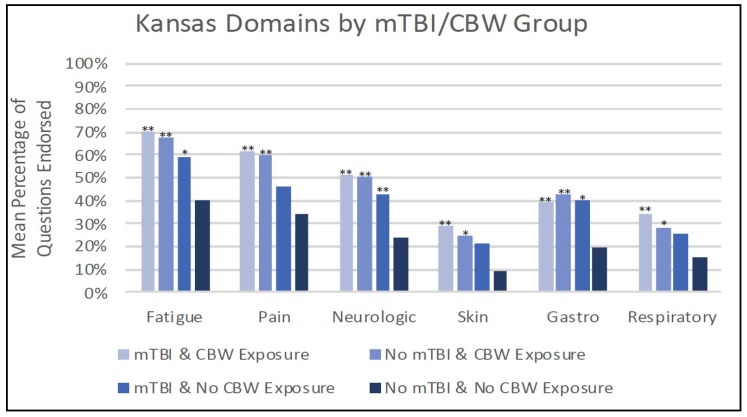
Kansas symptom domains by mTBI and CBW groups. * *p* < 0.05, ** *p* < 0.005 for comparison with reference group (no mTBI & no CBW exposure), mTBI = mild traumatic brain injury, CBW = chemical/biological weapons.

As described above, all of the veterans with both mTBI and CBW exposure met the criteria for GWI, as defined by the Kansas criteria (Table 2). Upon further examination, it was evident that those veterans with both exposures (mTBI and CBW) endorsed symptoms in all six symptom domains (fatigue, pain, neurological, skin, gastrointestinal, and respiratory) and significantly more symptoms in each domain (Figure 2). This profile was also seen in the exposure group with CBW only. The exposure group defined as mTBI-only endorsed a significantly higher percent of symptoms in the fatigue, neurological, and gastrointestinal symptom domains compared to the unexposed group.

### 3.6. Association between Exposure Groups and Chronic Health Conditions

Elevated unadjusted odds ratios, reaching statistical significance, were found in four chronic conditions and across all three exposure groups when compared with the non-exposed group (Table 3). Increased odds of having respiratory allergies or sinus problems, repeated trouble with neck, back or spine, memory loss, and depression was seen in the CBW exposure vs. no exposure groups, the mTBI exposure vs. no exposure, and the dual mTBI and CBW exposure vs. no exposure group. Two additional chronic conditions had statistically significant elevated odds ratios in both the CBW exposure vs. no exposure and the dual mTBI and CBW exposure vs. no exposure groups. These two chronic conditions were chronic fatigue syndrome and irritable bowel syndrome. The remaining eight health outcomes with statistically significant elevated odds ratios were only seen in the dual mTBI and CBW exposure groups and included asthma, high sugar or diabetes, blindness or trouble seeing with either eye, chronic lung disease, chronic skin conditions, deafness or trouble hearing in either ear, chemical sensitivity, and “other” gastrointestinal disorders. In summary, a total of 12 chronic conditions had statistically significant elevated odds ratios in the dual mTBI and CBW exposure group, six chronic conditions for the group exposure to CBW exposures only, and four chronic conditions for those with mTBI exposures only during the war.

## 4. Discussion

The current study examined a multiple hit hypothesis (mTBI and CBW) in 1990–1991 GW veterans in relation to GWI and chronic medical conditions. To our knowledge, this is the first paper to examine exposure to both mTBI and a neurotoxicant (CBW) in relation to GWI. In our previous studies, we investigated the multiple hit hypothesis using two neurotoxicants known to be frequent in the GW, pesticides, and pyridostigmine bromide (PB) pills. We found a strong synergistic effect of these mixed exposures on GWI and cognitive outcomes [1]. Given the brevity of the GW ground war, mTBI has not been investigated as a primary factor of GWI until recently. In our recent work with the Ft. Devens Cohort, veterans with mTBI had increased rates of GWI as determined by Centers for Disease Control (CDC) and Kansas GWI criteria [16,17]. These studies also showed that the increased number of mTBIs was associated with higher number of total symptoms reported [16]. However, the timing of the mTBI was not available for the Ft. Devens Cohort studies, leaving open the question of whether the proximity of the mTBI to CBW exposure had an impact on the rates of GWI and comorbid chronic health conditions.

Findings from the current study identified a surprisingly strong effect of timing of mTBI during the war and GWI in that those who experienced a mTBI during the war were statistically significantly more likely to endorse symptoms consistent with GWI than those who did not report mTBI during the war. Conversely, rates of mTBI in the GWI cases and controls did not differ before or after the war, indicating that the timing of the mTBI insult appeared to be critical to development of chronic health symptoms. These results add an important concept to consider when doing GWI research. This suggests that brain sequelae of mTBI as well as neurotoxicant induced brain changes, including white matter (WM) macro and microstructural integrity alterations [30,31,32], should be considered as prime candidates in the chronic CNS pathobiology of GWI.

The only other study reporting on mTBI rates in GW veterans found remarkably similar rates to ours with 47% of their GW veteran cohort and 46% of our GWIC cohort reporting a history of ever having an mTBI [18]. Chao also reported that only 7% of her sample as compared with 36% of our sample reported mTBIs sustained during the war. Chao did not find an association between the history of mTBI during the war and GWI, however the study did find that those with mTBI during the war had increased chronic symptoms. The lack of association with GWI could be influenced by the small sample size of veterans who sustained a mTBI during the war (*n* = 7). However, our data showed an association between mTBI such that nearly half of our cases and none of our controls reported a mTBI during the war. The differences in respective rates of mTBIs during the war may reflect the military occupational specialties (MOS) of our cohort. Our multi-site GWI consortium was more representative of the whole GW veteran cohort because participants were recruited from three sites across the country (Boston, Houston, and Miami), had varied MOS, and represented every branch of military service, making them representative of all deployed GW veterans.

The most relevant findings of this study included the replication of results from our previous work that mTBI is associated with increased rates of GWI. In relation to comorbid health conditions, findings emerged that confirm our hypothesis of a synergistic effect, with increased odds ratios for those with both mTBI and CBW on 12 of the total medical conditions. In essence, the “two hit” group showed more than double the reporting of chronic conditions than the “one hit” groups. Correspondingly, in this study, we report that GW veterans with both mTBI and CBW had significantly increased health symptoms across all six Kansas GWI criteria domains, including fatigue, pain, neurologic, skin, gastrointestinal, and respiratory symptoms [10]. These results suggest that more than the CNS is affected in these groups.

However, confirmation of our original hypothesis that multiple exposures in this two hit model (mTBI and CBW) are consistent with increased rates of GWI was not found in that the rate of GWI did not differ between those with multiple versus single hits. The difference in the rates of GWI may have been obscured because individuals with mTBI and no CBW may have had other exposures, including sarin, pesticides, and PB pills that were not accounted for in these analyses. These additional neurotoxicant exposures could have influenced the rates of GWI in those without CBW.

While the current study cannot confirm the etiology for GWI symptoms, several studies suggest that the neurotoxicant exposures experienced in war can lead to an increased immune response, not only in the brain, but throughout the body [33,34,35,36]. Likewise, TBIs can also lead to an increased neuroinflammatory state by priming the immune cells of the brain—microglia and astrocytes—to respond longer and stronger after each additional encounter with foreign or “danger” associated molecular patterns (DAMPS) from damaged or apoptotic neural cells [37,38,39,40,41]. This chronic inflammatory overactivation has now been termed post-inflammatory brain syndrome (PIBS) [42]. GWI sequelae fits this profile and can be considered a result of PIBS depending on the individual veterans’ “danger” exposure history (i.e., multiple toxicant or mTBI exposures or both).

Consistent with the literature on multiple hits, when more than one exposure occurs, more CNS dysfunction and other body systems are affected [16,43]. In this scenario, damaged or apoptotic cells from the brain can signal into the periphery and vice-versa from a leaky blood brain barrier (BBB) or gut-brain axis [36,44,45]. Future studies should further investigate the pathobiology of this multiple hit hypothesis.

Like all studies, this study had several limitations worth mentioning. First, the use of a relatively small cohort of GW veterans who self-reported exposures during the war suggests that further study is warranted in larger study samples. Second, the mTBI and CBW exposures were self-reported and the chronic conditions were not confirmed by medical records. However, the veterans did endorse that their chronic conditions had been diagnosed by a physician. Although the sample size of the groups was modest, it was more representative than other cohorts reporting on neurotoxicant and mTBI outcomes published to date in that it represented GW veterans from all branches of the military and three study sites around the country. Third, we examined associations on 18 outcome measures across four exposure groups, and, given our limited sample size, we did not control for multiple comparisons. However, we found elevated risks for 12 out of 18 outcomes in the multiple hit exposure group, which was far more than would be expected by chance. There remain questions about the timing of the mTBI and CBW exposures that could not be answered in this study, such as whether mTBI exposure increases CBW toxicity. It was also unclear whether mTBI is specific to the multiple hits needed for chronic conditions or if it represented a proxy for other exposure combinations during deployment.

## 5. Conclusions

The current study examined the relation of the separate and combined effects of exposure to mTBI and CBW in 1991 GW veterans. The findings from this study suggest that both exposure to mTBI and CBW are associated with the development of GWI and multiple chronic health conditions and that combined exposure appears to lead to higher risk of chronic health effects.

## Figures and Tables

**Table 1:** Population characteristics.

Total Sample = 125	
Age, years: Mean (SD)	51.5 (6.0)
Females: *n* (%)	19 (15.2%)
Race: *n* (%)	
Black/African America *n*	18 (14.4%)
White/Caucasian	93 (74.4%)
Other/Multiracial	14 (11.2%)
Hispanic or Latino: *n* (%)	12 (9.6%)
Education: *n* (%)	
Grade 12	8 (6.4%)
GED	1 (0.8%)
HS plus technical training	13 (10.4%)
Associate/2 years college	29 (23.2%)
Some college	17 (13.6%)
Bachelors	22 (17.6%)
Advanced degree	35 (28.0%)
Military Branch: *n* (%)	
Army	84 (67.2%)
Marines	13 (10.4%)
Navy	20 (16.0%)
Air Force	8 (6.4%)
Current PTSD: *n* (%) [*n* = 91]	34 (37.4%)
GWI Case (Kansas): *n* (%)	104 (83.2%)
Self-reported mTBI, during deployment: *n* (%)	44 (35.2%) [Mean SD among those with: 2.0 (1.4)]
Self-reported mTBI, prior to deployment: *n* (%)	46 (36.8%) [Mean SD among those with: 2.5 (2.3)]
Self-reported mTBI, after deployment: *n* (%)	33 (26.4%) [Mean SD among those with: 2.4 (2.0)]
Self-reported chemical/biological exposure: *n* (%)	59 (47.2%)

Note: GED = General Equivalency Degree, HS = high school, PTSD = post-traumatic stress disorder, mTBI = mild traumatic brain injury, GWI = Gulf War Illness.

**Table 2:** Demographic characteristics by mTBI and chemical/biological weapon (CBW) exposure categories.

Variable	*n* Mean (SD)	mTBI & CBW	mTBI & No CBW	No mTBI & CBW	No mTBI & No CBW	*p*-Value
Age	*n*	26	18	33	48	0.50
	Mean (SD)	50.1 (6.0)	51.3 (5.1)	51.4 (4.9)	52.4 (7.0)	
Gender	Female, *n* (%)	5 (19.2%)	1 (5.6%)	9 (27.3%)	4 (8.3%)	0.07
	Male, *n* (%)	21 (80.8%)	17 (94.4%)	24 (72.7%)	44 (91.7%)	
Race	Black/African American, *n* (%)	4 (15.4%)	5 (27.8%)	2 (6.1%)	7 (14.6%)	0.49
	White/Caucasian, *n* (%)	18 (69.2%)	11 (61.1%)	28 (84.8%)	36 (75.0%)	
	Other/Multiracial, *n* (%)	4 (15.4%)	2 (11.1%)	3 (9.1%)	5 (10.4%)	
Hispanic or Latino	Yes, *n* (%)	6 (23.1%)	1 (5.6%)	0 (0.0%)	5 (10.4%)	0.03
	No, *n* (%).	20 (76.9%)	17 (94.4%)	33 (100.0%)	43 (89.6%)	
Education	Grade 12, *n* (%)	0 (0.0%)	2 (11.1%)	0 (0.0%)	6 (12.5%)	0.50
	GED, *n* (%)	0 (0.0%)	0 (0.0%)	1 (3.0%)	0 (0.0%)	
	HS plus technical training, *n* (%)	3 (11.5%)	4 (22.2%)	2 (6.1%)	4 (8.3%)	
	Associate/2 years college, *n* (%)	6 (23.1%)	5 (27.8%)	9 (27.3%)	9 (18.8%)	
	Some college, *n* (%)	5 (19.2%)	2 (11.1%)	4 (12.1%)	6 (12.5%)	
	Bachelors, *n* (%)	6 (23.1%)	2 (11.1%)	6 (18.2%)	8 (16.7%)	
	Advanced degree, *n* (%)	6 (23.1%)	3 (16.7%)	11 (33.3%)	15 (31.3%)	
Current PTSD (*n* = 91)	Yes, *n* (%)	10 (50.0%)	2 (22.2%)	8 (33.3%)	14 (36.8%)	0.49
	No, *n* (%)	10 (50.0%)	7 (77.8%)	16 (66.7%)	24 (63.2%)	
GWI case (Kansas)	Yes, *n* (%)	26 (100.0%)	18 (100.0%)	30 (90.9%)	30 (62.5%)	<0.01
	No, *n* (%)	0 (0.0%)	0 (0.0%)	3 (9.1%)	18 (37.5%)	
Military Branch	Army, *n* (%)	18 (69.2%)	11 (61.1%)	26 (78.8%)	29 (60.4%)	0.68
	Marines, *n* (%)	3 (11.5%)	2 (11.1%)	4 (12.1%)	4 (8.3%)	
	Navy, *n* (%)	3 (11.5%)	4 (22.2%)	2 (6.1%)	11 (22.9%)	
	Air Force, *n* (%)	2 (7.7%)	1 (5.6%)	1 (3.0%)	4 (8.3%)	

mTBI = mild traumatic brain injury; CBW = chemical/biological weapons, GED = General Equivalency Diploma, HS = high school, PTSD = post-traumatic stress disorder, GWI = Gulf War Illness.

**Table 3:** Unadjusted logistic regression analysis examining CBW exposure, mTBI exposure, and mTBI + CBW.

Variable	CBW Exposure Only vs. Neither	mTBI Exposure Only vs. Neither	mTBI + CBW Exposure vs. Neither
	OR (95% CI)	OR (95% CI)	OR (95% CI)
High Blood Pressure	0.80 (0.32,1.99)	2.80 (0.90,8.72)	0.88 (0.33,2.32)
Heart Attack or MI	1.47 (0.09,24.35)	2.76 (0.16,46.70)	1.88 (0.11,31.35)
Asthma ^1^	5.11 (0.96,27.13)	2.87 (0.37,22.13)	6.90 (1.28,37.18) *
High sugar/diabetes ^1^	2.07 (0.43,9.92)	4.28 (0.85,21.49)	4.50 (1.02,19.81) *
Respiratory allergies or sinus problems ^1,2,3^	5.88 (2.16,16.00) *	5.42 (1.67,17.61) *	6.93 (2.37,20.26) *
Arthritis, rheumatism or gout	1.54 (0.59,3.99)	2.69 (0.88,8.27)	1.68 (0.61,4.64)
Repeated trouble with neck, back or spine ^1,2,3^	3.30 (1.30,8.35) *	4.86 (1.52,15.51) *	10.20 (3.21,32.44) *
Blindness or trouble seeing with either eye ^1^	3.33 (0.77,14.44)	4.29 (0.85,21.50)	7.94 (1.92,32.88) *
Chronic lung disease ^1^	4.70 (0.47,47.30)	9.40 (0.91,97.25)	20.89 (2.44,179.09) *
Chronic skin conditions ^1^	2.93 (0.99,8.62)	2.25 (0.61,8.32)	6.83 (2.25,20.78) *
Memory loss ^1,2,3^	5.67 (2.05,15.65) *	3.97 (1.22,12.95) *	8.39 (2.50,28.21) *
Deafness or trouble hearing in either ear ^1^	1.62 (0.57,4.67)	2.76 (0.84,9.09)	4.33 (1.51,12.46) *
Depression ^1,2,3^	2.64 (1.05,6.61) *	4.40 (1.39,13.96) *	4.16 (1.51,11.44) *
Current PTSD [N = 91]	0.86 (0.29, 2.51)	0.49 (0.09, 2.69)	1.71 (0.57, 5.13)
Chemical sensitivity ^1^	2.62 (0.83,8.27)	3.50 (0.95,12.85)	8.17 (2.58,25.83) *
Chronic Fatigue Syndrome ^1,2^	5.17 (1.96,13.65) *	2.69 (0.85,8.48)	9.13 (3.05,27.35) *
Irritable Bowel Syndrome ^1,2^	4.71 (1.82,12.22) *	1.71 (0.55,5.37)	4.31 (1.56,11.88) *
Other GI disorders ^1^	1.46 (0.55,3.87)	1.12 (0.33,3.81)	3.40 (1.24,9.36) *

* = statistically significant. ^1^ = statistically significant difference between multiple exposure (mTBI + CBW) and no exposure. ^2^ = statistically significant difference between chemical/biological-weapons-only exposure and no exposure. ^3^ = statistically significant difference between mTBI-only exposure and no exposure. mTBI = mild traumatic brain injury; CBW = chemical/biological weapons; MI = myocardial infarction; GI = gastrointestinal.
